# Qualitative exploration of manual dental records in the digital era using sociotechnical systems theory: insights from a teaching dental institution

**DOI:** 10.12701/jyms.2026.43.9

**Published:** 2026-01-05

**Authors:** Syuwari Azhar Azman, Sulhi Abidin, Galvin Sim Siang Lin, Mohd Haikal Muhamad Halil

**Affiliations:** 1 Department of Restorative Dentistry, Kulliyyah of Dentistry, International Islamic University Malaysia, Pahang, Malaysia; 2 Department of Prosthodontics, Kulliyyah of Dentistry, International Islamic University Malaysia, Pahang, Malaysia

**Keywords:** Computerized medical records systems, Dental records, Dentistry

## Abstract

**Background:**

Dental records are essential repositories of patient information that support diagnosis, treatment planning, continuity of care, and medicolegal accountability, making them a fundamental component of safe, effective, and transparent dental practices. This study aimed to explore the perceptions and experiences of academic and nonacademic clinical staff regarding a paper-based patient record system at a Malaysian public dental institute.

**Methods:**

A qualitative phenomenological design underpinned by the sociotechnical systems theory was employed. Purposive sampling recruited 20 full-time staff (10 academic, 10 nonacademic) with at least 1 year of experience using the manual record system and no prior training in electronic dental records. Semi-structured, one-on-one interviews were conducted between May 2025 and October 2025, audio-recorded, transcribed verbatim, and analyzed using Braun and Clarke’s thematic analysis. Rigor was enhanced through independent coding, member checking, reflexive journaling, and adherence to the Consolidated Criteria for Reporting Qualitative Research checklist.

**Results:**

Four main themes were identified: (1) inefficiency and accessibility challenges; (2) accuracy, legibility, and record integrity; (3) continuity of care and patient safety risks; and (4) desire for digital transition and system improvements.

**Conclusion:**

Although manual paper-based dental records remain central to documentation in teaching dental institutions, they present growing inefficiencies and safety concerns, highlighting the need for sociotechnical-informed strategies that align human processes, technology, and institutional support to enhance record-keeping and patient care.

## Introduction

Dental records are a cornerstone of clinical dentistry and serve as a comprehensive repository of patient information. They encompass personal details, medical and dental histories, diagnoses, treatment plans, and outcomes, thereby supporting continuity of care across multiple encounters [1]. Traditionally, these records have been paper-based and include handwritten notes, radiographs, diagrams, and supporting documentation, which together provide a chronological account of a patient’s oral health journey. Beyond their clinical function, records also act as a vital communication tool not only among dental professionals but also between dentists and patients, thereby reinforcing accountability, transparency, and trust in the therapeutic relationship [2,3]. The significance of maintaining accurate records extends across both clinical and legal domains. Clinically, they facilitate effective diagnosis, evidence-based treatment planning, and the long-term monitoring of patient progress [4]. Records enable clinicians to review previous interventions, adapt treatment strategies, and provide comprehensive patient care. Legally, they constitute essential documentation in cases of complaints, disputes, or litigation [5]. A well-maintained record demonstrates adherence to standards of care and protects practitioners and patients [3,6]. For these reasons, dental records are not regarded as administrative formalities but as central to the quality, safety, and accountability of dental practice.

For many years, healthcare facilities, especially those in resource-constrained environments, have relied on manual record-keeping systems [7]. These systems function without the need for digital infrastructure, electricity, or internet connectivity, making them especially practical in rural or under-resourced areas. Furthermore, paper-based systems are inherently immune to digital threats such as hacking or system crashes, which makes them appear safer from a cybersecurity perspective [8]. These attributes may have contributed to the persistence of manual record-keeping despite advances in technology. Despite their longstanding utility, manual or paper-based dental records present considerable challenges. They are physically prone to wear and tear, environmental damage, and accidental loss [9], resulting in incomplete patient histories and potential compromises in the quality of care [10]. Additionally, manual systems lack interoperability, hindering the seamless exchange of information across departments and between healthcare providers [11]. In busy clinical or academic environments, the logistical burden of retrieving and archiving physical records may further increase the administrative workload and disrupt workflow [12,13].

Globally, the digitalization of healthcare documentation has been proposed as a solution to many of these challenges. Electronic dental record (EDR) systems promise improved data accessibility, accuracy, and long-term preservation, while supporting delivery of integrated care and institutional efficiency [10,13]. However, the success of digital transformations is rarely determined by technology alone. Organizational readiness, user acceptance, and perceptions among key stakeholders play pivotal roles in determining whether digital record systems are effectively adopted and sustained [7,14]. In dental education settings, this involves not only academic staff who provide clinical supervision, but also nonacademic personnel who manage patient registration, billing, and record maintenance. Their collective experiences and attitudes shape their daily record-keeping practices and influence the feasibility of transitioning from manual to electronic systems. Despite the increasing emphasis on digital transformation within healthcare [15], there is a notable paucity of recent empirical studies examining the lived experiences of dental school staff who continue to rely on manual paper-based record systems. Qualitative inquiry has been shown to provide deep insights into lived experiences and contextual factors in dental education settings [16].

Understanding these perceptions is crucial as they reveal both the operational realities of current systems and institutional readiness for digital change. Insights from staff directly involved in patient care and administrative coordination can guide context-sensitive strategies to implement EDR systems that enhance rather than disrupt clinical and educational workflows. Accordingly, the present qualitative study aimed to explore the perceptions of academic and nonacademic clinical staff at a Malaysian public dental school regarding the existing paper-based patient record system. By capturing staff experiences and viewpoints, this study offers valuable insights into the human and contextual dimensions of dental record management and provides evidence-based approaches toward future digital transformation in dental education.

## Methods

**Ethics statement:** The study was approved by the International Islamic University Malaysia Research and Ethics Committee with registration number IREC 2024-375. Informed consent was obtained from all participants included in the study. The study protocol conforms to the ethical guidelines of the 1975 Declaration of Helsinki. All subjects’ rights were protected, and all data were kept confidential.

This study followed the Consolidated Criteria for Reporting Qualitative Research 32-item checklist to ensure transparency and rigor in the study design, data collection, analysis, and reporting [17,18].

### 1. Theoretical framework

The present study is underpinned by sociotechnical systems theory as a sensitizing theoretical framework. This theory posits that organizational outcomes are shaped by interactions between people, processes, and technology [19]. This study provides a lens for interpreting how human factors (staff roles and experiences) and organizational systems (manual records) interact to influence efficiency, accuracy, and patient safety.

### 2. Study context

This study was conducted at the Kulliyyah of Dentistry, International Islamic University Malaysia. The faculty operate within this teaching and clinical institution, providing dental care to patients from the local community while simultaneously training undergraduate and postgraduate dental students. Patient records are currently maintained using a manual paper-based system that includes handwritten notes, diagnostic charts, treatment plans, radiographs, and other supporting documentation. Academic staff (clinicians and educators) and nonacademic staff (administrative and record officers) are actively involved in managing and utilizing these records daily. This setting provides a natural context in which to holistically explore the experiences and challenges faced by staff members when dealing with manual patient dental records.

### 3. Study design

A qualitative phenomenological design situated within an interpretivist paradigm was employed. Interpretivism assumes that knowledge is socially constructed and that meanings are co-created through interactions between participants and researchers [20,21]. Phenomenology was selected because it allows for a deep exploration of participants’ lived experiences with manual record-keeping, capturing how they perceive, interpret, and make sense of its advantages, limitations, and practical implications.

### 4. Sampling and recruitment

Purposive sampling was used to recruit participants who were directly engaged with the patient record system. Both academic and nonacademic staff members were included to ensure maximum variation and capture diverse perspectives. The inclusion criteria were as follows: (1) employed as full-time clinical staff members at the dental school, (2) having at least 1 year of experience using the manual record system, and (3) having no prior training or experience in EDR systems. Staff members with minimal or no direct interaction with patient records were excluded.

Departmental announcements and direct invitations from the research team facilitated recruitment. Twenty participants were enrolled comprising 10 academic staff (lecturers and clinical supervisors) and 10 nonacademic staff (administrative officers, record clerks, and support staff). This balanced composition enabled us to explore both clinical and administrative dimensions of record-keeping practices. Data collection continued until thematic saturation was reached and no new significant themes emerged from additional interviews.

### 5. Data collection

Semi-structured, one-on-one interviews were conducted between May 2025 and October 2025. An interview guide was developed, and the content was validated by two senior experts with extensive experience in dental research. The guide comprised open-ended questions designed to elicit rich narratives, while allowing flexibility for the interviewer to probe and explore emergent topics in depth. Example prompts include the following:

*“Can you describe your experience with the manual patient record system?”*

*“What challenges do you face in retrieving or recording information?”*

*“How do manual records affect your daily workflow and patient care?”*

*“What improvements or alternatives would you suggest?”*

While the primary focus was participant experiences with the existing manual record system, the interview guide also included an exploratory prompt inviting reflections on potential improvements or alternative systems. This allowed participants to discuss their perceived readiness and expectations for digital transformation where relevant.

Interviews were conducted in private meeting rooms within the institution or via secure online platforms (e.g., Zoom [Zoom Video Communications, San Jose, CA, USA]), depending on participant preference. Each interview lasted for 20 to 30 minutes. All interviews were audio-recorded with participant consent and transcribed verbatim. Field notes were kept after each interview to document contextual observations, nonverbal cues, and immediate reflections. No prior relationships existed between the researcher and the participants before the interviews. An iterative process was used in which early insights from the initial interviews informed refinements for subsequent questioning.

### 6. Data analysis

Thematic analysis was conducted following Braun and Clarke’s six-phase framework [22]. First, the transcripts were read multiple times for familiarization. Second, initial codes were generated inductively to capture meaningful units of text. Third, the codes were collated into categories and potential themes. Fourth, the themes were reviewed, compared, and refined to ensure coherence and distinction. Fifth, the themes were defined and named to reflect their conceptual essence. Finally, the findings were synthesized into four overarching themes supported by illustrative participant quotations. NVivo software (ver. 15.2.0; QSR International, Melbourne, Australia) was used to organize and manage the coding process.

To enhance the rigor, two researchers (SAA, SA) independently coded a subset of transcripts and compared their coding to establish consistency. Discrepancies were resolved through discussions with a third researcher (GSSL). Member checking was conducted on a sample of five participants who reviewed the preliminary themes to confirm that their interpretations reflected their perspectives. A thematic map was developed to visually depict the relationships between the themes.

### 7. Researcher characteristics and reflexivity

The first two researchers (SAA, SA) are male clinician-academics with doctoral qualifications in clinical dentistry, and they conducted all participant interviews. Both had received prior training and mentorship in qualitative interviewing and research design from the third researcher (GSSL) with extensive expertise in dental research and qualitative methodologies. While this background facilitated contextual understanding, it also posed a risk of bias. To mitigate this, the researchers maintained a reflexive journal documenting the assumptions, methodological decisions, and evolving interpretations throughout the study. Bracketing was employed to consciously set aside preexisting assumptions about digital systems. Peer debriefings were conducted among the research team members.

## Results

**Fig. 1.**
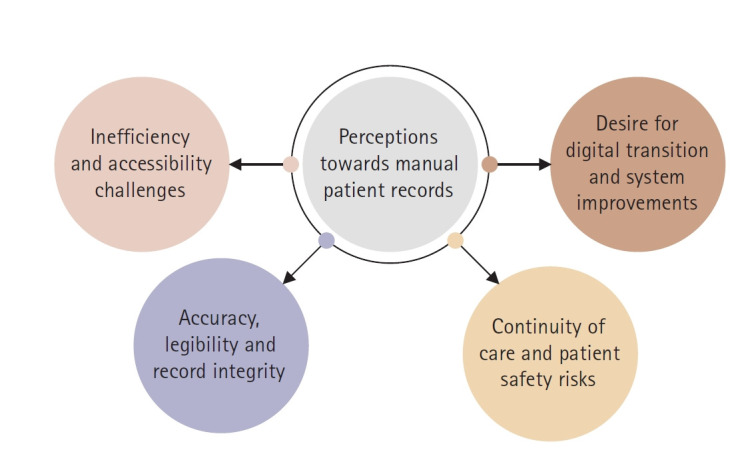
Thematic map illustrating the four key themes identified from the qualitative analysis of participants’ experiences with the manual dental record system.

Twenty participants were involved in one-on-one interviews to explore their perceptions of the manual record system. Saturation was reached at the 18th participant; however, two additional interviews were conducted to ensure confirmatory saturation and allow for consistency of emergent themes across different staff categories. Thematic analysis identified four themes (Fig. 1): (1) inefficiency and accessibility challenges; (2) accuracy, legibility, and record integrity; (3) continuity of care and patient safety risks; and (4) desire for digital transition and system improvements. Each theme is elaborated below with illustrative quotes.

### 1. Theme 1. Inefficiency and accessibility challenges

Participants consistently described the manual system as cumbersome and time-consuming, with inefficiencies evident in both clinical and administrative processes. The physical retrieval of folders, delays in patient registration, and frequent misplacement of files were recurring frustrations, particularly among the front-desk and clerical staff. These inefficiencies were perceived to impede workflow, increase patient waiting times, and generate fatigue among users. Example responses are indicated below.

*“…12 years experience here, tired of writing notes, difficult to manage...”* (participant [P] 12)

*“…really time-consuming because I need to find the patient’s folder...”* (P9)

*“(I) have to wait longer for the folder...”* (P4)

*“Main issue: missing files, delayed in searching…because there are too many folders”* (P11)

*“Difficulty in tracking past records. Misplacement of the folder is common...”* (P7)

*“…YES! difficult to trace the location of files…”* (P13)

### 2. Theme 2. Accuracy, legibility, and record integrity

In addition to physical accessibility, concerns were raised regarding the quality and reliability of handwritten documents. Participants highlighted that illegible handwriting, incomplete entries, and disorganized case notes often compromised data accuracy and interpretability, particularly during patient follow-ups or inter-staff communication. Some participants also expressed apprehension about the vulnerability of paper-based data to tampering or unauthorized access. Example responses are indicated below.

*“Case notes not in order. Difficult to interpret and don’t know what to do next...”* (P10)

*“Manual can be trouble to read, especially when handwriting is poor…”* (P20)

*“…information can be altered, stolen, and misused if using paper-based (clinical) notes…”* (P13)

### 3. Theme 3. Continuity of care and patient safety risks

The participants widely recognized that missing or incomplete records could have direct clinical consequences. The manual system was believed to interrupt the continuity of care, delay treatment, and limit the ability of practitioners to make informed decisions. In several accounts, participants described their inability to retrieve prior treatment histories as a key barrier to providing safe and efficient care. Example responses are indicated below.

*“If the folder is discarded, it will be very difficult to trace back patients’ treatment history...”* (P10)

*“Slow the process of screening or treatment as doctors wait for files…”* (P15)

*“…affected the patients, 100% lead to delay in treatment if it (patient’s case note) is missing…”* (P17)

### 4. Theme 4. Desire for digital transition and system improvements

Across all interviews, there was a strong consensus on the need for modernization and digital transformation. Participants viewed digital record systems as a solution to current inefficiencies, possibly offering faster retrieval, enhanced accuracy, and improved data security. The prospect of a *“paperless”* environment was framed as both desirable and necessary for aligning with contemporary healthcare practices. Example responses are indicated below.

*“Yes. It is time to transform to digital data…definitely more reliable”* (P1)

*“Paperless and reduce the missing folder issues...digital (record system) can ensure that patient case notes are up to date”* (P3).

*“Yes, I agree as it (digital record system) is more reliable and accurate, as paper might go missing…”* (P12)

*“Transfer the folder storage room to another building is tiring, but if digital, it would be great and more efficient...”* (P20)

## Discussion

This qualitative study explored the perceptions and experiences of academic and nonacademic staff regarding the paper-based patient record system at a Malaysian dental school. Using sociotechnical systems theory as an interpretive lens, the findings revealed that inefficiency, inaccessibility, and issues of accuracy and legibility within the manual record system have direct implications for continuity of care, patient safety, and overall organizational effectiveness. Collectively, the present themes illuminate the complex interplay between human, organizational, and technological factors that shape the record-keeping environment in dental education settings.

Participants described the manual record system as time-consuming, tiring, and frustrating, particularly when retrieving folders, registering patients, or locating misplaced files. These experiences mirror those of previous studies showing that manual documentation systems may disrupt clinical workflow, increase waiting times, and contribute to staff stress and burnout [9,10,23]. The time spent searching for records or rewriting lost notes constitutes lost clinical hours that could otherwise be devoted to direct patient care. From a sociotechnical standpoint, this theme illustrates a clear mismatch between the technical subsystem (paper records, storage rooms, and filing routines) and the social subsystem (staff who must deliver timely care and manage patient flow). Administrative staff carry an additional burden of tracking and reorganizing misplaced files, which strains institutional resources and duplicates effort [24]. These inefficiencies inevitably spill over to patients, and longer waiting times owing to delayed record retrieval are associated with reduced satisfaction and perceptions of poor service quality. Thus, the frustrations expressed by participants represent not only a personal burden but also a systemic issue that undermines organizational performance and patient experience.

Concerns emerged regarding the reliability and quality of paper-based records. Participants highlighted difficulties interpreting disordered notes and illegible handwriting, aligning with the evidence that poor documentation quality in manual systems can lead to miscommunication and clinical errors [25]. Beyond accuracy, participants raised concerns about the integrity and security of manual records. Although paper-based systems are sometimes perceived as less vulnerable to cyberattacks, they are highly susceptible to wear and tear, loss, and unauthorized alterations [9]. Viewed through sociotechnical systems theory, these issues reflect an information-integrity gap at the interface of human behavior and technical processes. Handwriting practices, filing habits, and storage conditions interact to produce documentation that is sometimes illegible, incomplete, or insecure. Legibility errors have been linked to adverse events in clinical practice, and the absence of robust audit trails in paper records weakens an institution’s ability to defend care practices in the event of a complaint or legal dispute [4,26,27]. In other words, the technical subsystem, as currently configured, fails to reliably support the social goals of clear communication, accountability, and safe practices.

Participants were particularly concerned about the implications of missing or incomplete records for the continuity of care and patient safety. Treatment progress may be delayed and, in some cases, appointments had to be postponed or cancelled at the last minute. This was especially problematic for patients who traveled long distances or took annual leave to attend appointments, leading to frustration and a perception of poor service [28,29]. Missing or inadequate records can also have medicolegal implications; failures in record-keeping expose institutions to potential malpractice claims when there is insufficient documentation to demonstrate appropriate care [12]. From a sociotechnical perspective, Theme 3 illustrates how breakdowns in the technical subsystem (e.g., folder loss, storage failures, and poor tracking mechanisms) directly erode the social subsystem’s ability to provide coordinated and safe care. The participants’ accounts suggest that repeated difficulties in locating patient folders or interpreting incomplete documentation may gradually normalize the use of workarounds. Under such conditions, healthcare workers may adopt compensatory behaviors, such as relying on memory, recording information retrospectively, or abbreviating documentation [30,31]. Although understandable, these adaptations increase the likelihood of incomplete or inaccurate records, thereby increasing patient safety risks. Thus, technical deficiencies indirectly shape human behavior, transforming system-level inefficiencies into clinical vulnerabilities. Furthermore, this fragmentation affects not only patient care but also educational oversight because clinical supervisors rely on accurate records to monitor student performance and clinical outcomes.

Theme 4, *“Desire for digital transition and system improvements,”* can be understood as a cumulative response to the inefficiencies, documentation problems, and safety concerns described above. Human errors in record-keeping, including incomplete documentation, are exacerbated by cognitive fatigue, insufficient training, and psychological overload among healthcare providers [32,33]. The participants described the burden of experiencing a loss of records, wasted time searching for folders, and physical fatigue from relocating files between locations. Moreover, the participants anticipated several benefits of digital record systems: faster access to patient data, reduced expenditure on paper and physical storage, and opportunities to repurpose storage spaces for other institutional needs. These expectations are consistent with previous evidence that digital systems can improve the efficiency, reliability, and security of record-keeping, thereby enhancing the quality of patient care [13-15]. Sociotechnical systems theory offers a useful way to interpret this theme. Participants are not simply asking for “more technology” but for a better alignment between technology, workflow, and organizational support. Their readiness and attitudes toward digitalization suggest that the social subsystem is primed for change, but successful implementation depends on careful joint optimization of social and technical elements. Although the participants in the present study appeared to hold generally positive attitudes toward digitalization, it remains uncertain whether such optimism would persist once an EDR system is implemented, given that no participant had prior experience using one. Consequently, the participants’ perceptions reflect anticipated benefits rather than lived experiences. The transition from paper-based to electronic systems is often accompanied by unexpected challenges, including increased documentation workloads, learning curves, and workflow disruptions [8,10,15]. Successful digital transformation also requires substantial investments in infrastructure, user training, and technical support. Thus, acknowledging this expectation, the reality gap provides a more balanced interpretation of staff readiness and highlights the importance of structured training, phased implementation, and organizational support.

A key strength of this study was the inclusion of both academic and nonacademic staff, offering a holistic view of the dental record system from multiple functional perspectives. The use of phenomenological design allowed for a deep exploration of lived experiences, while the application of sociotechnical systems theory provided a structured interpretive lens to understand the interdependence between human, organizational, and technological elements. Rigor was further enhanced through methodological triangulation, member checking, and reflexivity among researchers. However, this study has several limitations that must be acknowledged. As this was a single-institution study, the findings may not be directly applicable to other dental schools with different administrative structures or technological infrastructure levels. This study also relied on self-reported experiences, which may have been influenced by recall bias or social desirability. Despite these limitations, this study provides a foundational understanding of staff experiences that can inform future research and policy development. Future studies should explore the perspectives of dental students and patients, examine comparative experiences following EDR implementation, or employ mixed method approaches to quantitatively evaluate workflow efficiency and user satisfaction. Future digital transformation efforts should also consider the ethical and professional dimensions of technology integration [34].

In conclusion, the present study revealed that, while manual paper-based dental records continue to serve as the backbone of documentation within current dental teaching institutions, they pose increasing challenges related to efficiency, legibility, and patient safety. The lived experiences of academic and nonacademic clinical staff highlight systemic inefficiencies that impede clinical workflow and administrative coordination. Through the lens of sociotechnical systems theory, these issues reflect a misalignment between human processes and technical infrastructure. Importantly, the participants demonstrated readiness for digitalization and optimism toward a potential transition to EDR systems. However, successful implementation requires not only technological investment but also comprehensive staff training, workflow redesign, and institutional support to ensure a balanced integration of social and technical components. These insights can guide dental schools and policymakers in developing evidence-informed, context-sensitive digital transformation strategies that strengthen clinical efficiency, documentation quality, and patient-centered care.

Careful preparation is essential for institutions in similar contexts contemplating a digital transition. Practical measures include phased and role-specific staff training, the early involvement of clinical and administrative stakeholders, and robust data migration strategies to preserve record integrity and continuity of care. Institutions should also plan for transitional workflow disruptions and provide adequate technical and organizational support. Guided by sociotechnical principles, digital transformation must align technology with existing clinical practices, accountability structures, and patient safety goals to ensure its sustainable and effective adoption.
